# Machine Learning-based Correlation Study between Perioperative Immunonutritional Index and Postoperative Anastomotic Leakage in Patients with Gastric Cancer

**DOI:** 10.7150/ijms.72195

**Published:** 2022-07-04

**Authors:** Xuanyu Liu, Su Lei, Qi Wei, Yizhou Wang, Haibin Liang, Lei Chen

**Affiliations:** Department of General Surgery, Xinhua Hospital, Affiliated to Shanghai Jiao Tong University School of Medicine, No. 1665 Kongjiang Road, Shanghai 200092, China

**Keywords:** gastric cancer, anastomotic leakage, machine learning, immunonutritional index

## Abstract

**Backgrounds:** The immunonutritional index showed great potential for predicting postoperative complications in various malignant diseases, while risk assessment based on machine learning (ML) methods is becoming popular in clinical practice. Early detection and prevention for postoperative anastomotic leakage (AL) play an important role in prognosis improvement among patients with gastric cancer (GC).

**Methods:** This retrospective study included 297 patients with gastric cancer receiving gastrectomy between 2018 and 2021 in general surgery department of Xinhua Hospital. Perioperative clinical variables were collected to evaluate the predictive value for postoperative AL with 5 ML models. Then, AUROC was applied to identify the optimal perioperative clinical index and ML model for predicting postoperative AL.

**Results:** The incidence of postoperative AL was 6.1% (n=18). After the training of 5 ML classification models, we found that immunonutritional index had significantly better classification ability than inflammatory or nutritional index alone separately (AUROC=0.87 vs. 0.83, P=0.01; AUROC=0.87 vs. 0.68, P<0.01). Next, we found that support vector machine (SVM), one of the ML methods, with selected immunonutritional index showed significantly greater classification ability than optimal univariant parameter [CRP on postoperative day 4 (AUROC=0.89 vs.0.86, P=0.02)]. Also, statistical analysis revealed multiple variables with significant relevance to postoperative AL, including serum CRP and albumin on postoperative day 4, NLR and SII etc.

**Conclusion:** This study showed that perioperative immunonutritional index could act as an indicator for postoperative AL. Also, ML methods could significantly enhance the classification ability, and therefore, could be applied as a powerful tool for postoperative risk assessment for patients with GC.

## Introduction

Gastric cancer is currently the fourth most common malignant disease worldwide, presenting a particularly high morbidity rate in East Asian region 1. With the development of modern medical technology including surgical operation and perioperative healthcare, mortality rate after gastrectomy is becoming much lower than before. Currently, gastrectomy with D2 lymphadenectomy is the standard procedure for patients with advanced stage gastric cancer, and could provide possible curative treatment 2, 3. However, postoperative anastomotic leakage remains to be clinically concerning with an incidence of 2.1-14.6% and a mortality rate of up to 50% reportedly, which could cause prolonged hospital stay, increased overall cost, and even compromised long-term survival ^[4-7].^ Present methods for detecting postoperative AL mostly depend on laboratory examination and radiological diagnosis 8. However, in case of severe complications like AL, advanced medical treatment before clinically confirmed could seize the opportunity and greatly enhance curative effect 9, 10.

Thus, early-stage risk stratification for postoperative AL that allows medical intervention in advance shows great potential to improve overall in-hospital health care, which could lead to a more personalized treatment plan, less unnecessary examination or invasive operation, and therefore, better prognosis 11.

Recently, machine learning started to show great value in oncological researches, ranging from assessing efficacy of chemotherapy 12, predicting long-term prognosis 13-15 to making early diagnosis 16. Machine learning models could work on a variety of complex nonlinear data and integrate task-related input features, so as to construct a more robust model with better predictive performance for decision making 17. Nowadays, with digitalization of the medical records, in-patient big data becomes easier for doctors to record and access, including medical history, imaging reports, laboratory tests and other information. Much valuable information was buried with other redundant useless data. However, with the help of ML methods, the performance could be greatly improved with designed algorithms through empirical learning. ML could also improve data quality by feature screening, extraction, and dimensionality reduction etc., which is even more beneficial on big data 18, 19.

In this study, we hypothesized that perioperative immunonutritional index is clinically related to the occurrence of postoperative AL, and could predict the risk level with the help of trained machine learning models.

## Methods

### Study design

This retrospective study enrolled all the patients that meet the inclusion criteria in the department of general surgery of Xinhua Hospital from 2018 to 2021. The inclusion criteria contained following: adult patients (age ≥ 18), pathological diagnosis of gastric carcinoma and undergoing gastrectomy with lymphadenectomy. Exclusion criteria were patients with general or localized infection, pregnant, clinically unable to perform surgery, with mental illness that could obstruct follow-up study, taking immuno-suppressive drugs or with missing clinical data of any kind.

### Perioperative management

Once enrolled, all the patients underwent a thorough preoperative evaluation and data collection, including medical history, physical examination, laboratory and radiological examination, and anesthesia evaluation. Meanwhile, biopsies under gastroscopy were also needed to determine the pathological features and the depth of invasion. And, in order to rule out distant metastasis, Positron Emission Tomography-Computed Tomography (PET-CT) and abdominal enhanced CT were conducted when needed.

Open or laparoscopic gastrectomy with digestive tract reconstruction and lymph node dissection were performed following GC treatment guidelines, and all surgeries were performed by at least one experienced chief surgeon. The tumor staging was cataloged based on the American Joint Committee on Cancer TNM Staging System for Gastric Cancer (eighth edition).

All postoperative complication within 30 days of surgery were graded according to the Clavien-Dindo classification 20.

### Data collection and endpoint

Routine blood biochemistry examinations were taken within one week before operation, covering tests of leukocytes (10^9^/L), neutrophils (10^9^/L), platelets (10^9^/L), lymphocytes (10^9^/L), serum fibrinogen (g/L), serum hemoglobin (g/L), serum albumin (g/L). Other perioperative clinicopathological characteristics included: age, sex, height, weight, former medical history, duration of hospitalization, surgery procedures, tumor TNM staging and grading. Postoperative blood tests were collected on postoperative day (POD) 1, 4 and 7 after surgery, including tests of leukocytes (10^9^/L), neutrophils (10^9^/L), serum albumin (g/L), serum CRP.

In present study, the SII, PNI and NLR were calculated as follows: SII=N×P/L and NLR=N/L, where L, N, and P represent lymphocytes, neutrophils, and platelets respectively; PNI = serum albumin (g/L) + lymphocyte count × 5 (10^9^ /L).

The primary outcome was to find clinical relation and potential value between perioperative immunonutrition index and postoperative AL. The secondary outcome was to investigate the clinical value of ML models in risk assessment.

### Univariant statistical analysis

Univariant statistical analysis were conducted using R software (version 4.1.2). Continuous variables were described as mean ± standard deviation (SD) for normally distributed data and would be compared by *t*-test. While, other continuous variables were described as median and interquartile range (IQR), and would be compared by nonparametric tests. The performance of each variable was compared through area under the receiver operating characteristic curve (AUROC), using *pROC* package in R software. Statistically significance was considered as *P*<0.05.

### Classifier development and evaluation

**Figure 1:**
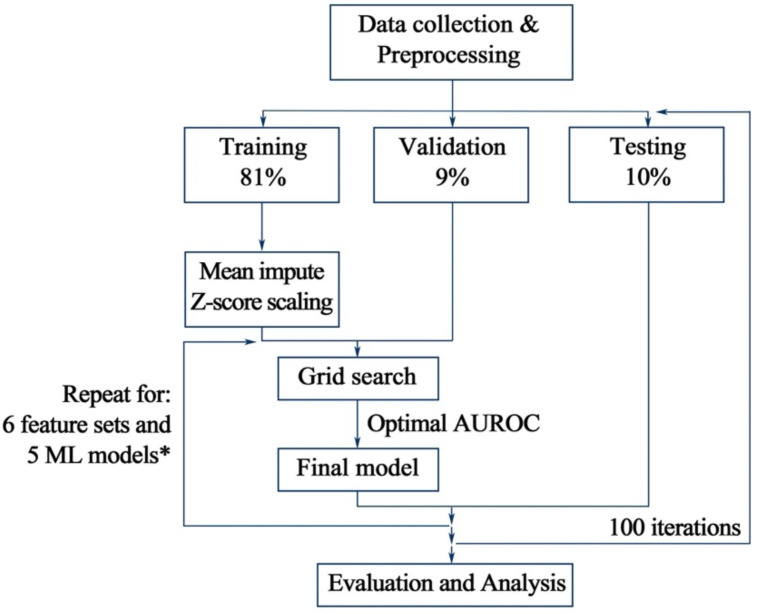
** Workflow for the classification methods and feature sets evaluation.** All data were split into 3 sets, including training, validation, and testing set. Five methods and six feature sets were tested and repeated for 100 iterations. Hyperparameter tuning would be performed with training and validation set. Then, combined and trained with optimal hyperparameter set, the highest AUROC (area under the receiver operating characteristic curve) for the validation set would be received. Afterwards, the final model would be applied in the testing set to verify its classification ability, the performance of which would be reported for each feature set. * Six feature sets include pre-operative, inflammatory, nutritional, immunonutritional, all and feature set with selected data. Five machine learning methods include *k*-NN, LR, SVM, RF and GB. *k*-NN: *k*-nearest neighbors; LR: logistic regression; SVM: support vector machine; RF: random forest; GB: gradient tree boosting.

We applied 6 types of feature sets and 5 different ML classification methods. Then, we adopted the training-validation-testing procedure, repeating 100 times each. In every single iteration, we divided all enrolled patients into three groups, including training set (81%), validation set (9%), or testing set (10%). The grid search was used in training and validation sets to find optimal hyperparameter for the classification model, and would be further verified through testing set. All procedures above were conducted with Python package scikit-learn. The detailed workflow was presented in Figure 1.

We evaluated the performance of ML models through AUROC with the *metrics.roc_auc_score* function from the Python package of scikit-learn. To compare the performance of different feature sets, a 2-sided paired sample *t* test was conducted, using the AUROCs of the test sets. The *t* test was conducted with *stats.ttest_rel* function within SciPy Python package.

Then, we evaluated the clinically relevant variables and measured the weight of contribution to the clinical outcome in order to achieve better improvement in ML performance. To that end, we conducted Shapley additive explanations (SHAP) to find clinical relevance of different variables in each feature set 21.

## Results

### Characteristics of patients

A total of 297 patients were eligible in this study, including 199 males and 98 females with a mean age of 63.5 years (± 10.2 years) and a mean BMI of 22.4 kg/m^2^ (± 3.2 kg/m^2^) (Table 2a). There were 196 (66%) patients who underwent laparotomic surgical procedure. Only a minority of patients (n=37, 12.5%) received intra-operative blood transfusion, and the median intra-operative blood loss was 150 ml (IQR, 100-200 ml). According to the eighth edition of the AJCC TNM staging system, this study was composed of 102 (34.3%) stage I, 67 (22.6%) stage II, 124 (41.8%) stage III and 4 (1.3%) stage IV patients.

Among all the patients included in this study, 18 patients (6.1%) altogether have shown symptoms and been clinically diagnosed with postoperative AL. Compared with those patients without postoperative AL, those who did were more likely to go through longer hospitalization and might experience more intra-operative blood loss (P<0.01). However, there were no significant differences in age, BMI or TNM staging between patients with or without postoperative AL (Table 2a and Table 2b).

### Univariant analysis of perioperative immunonutritional index

**Figure 2:**
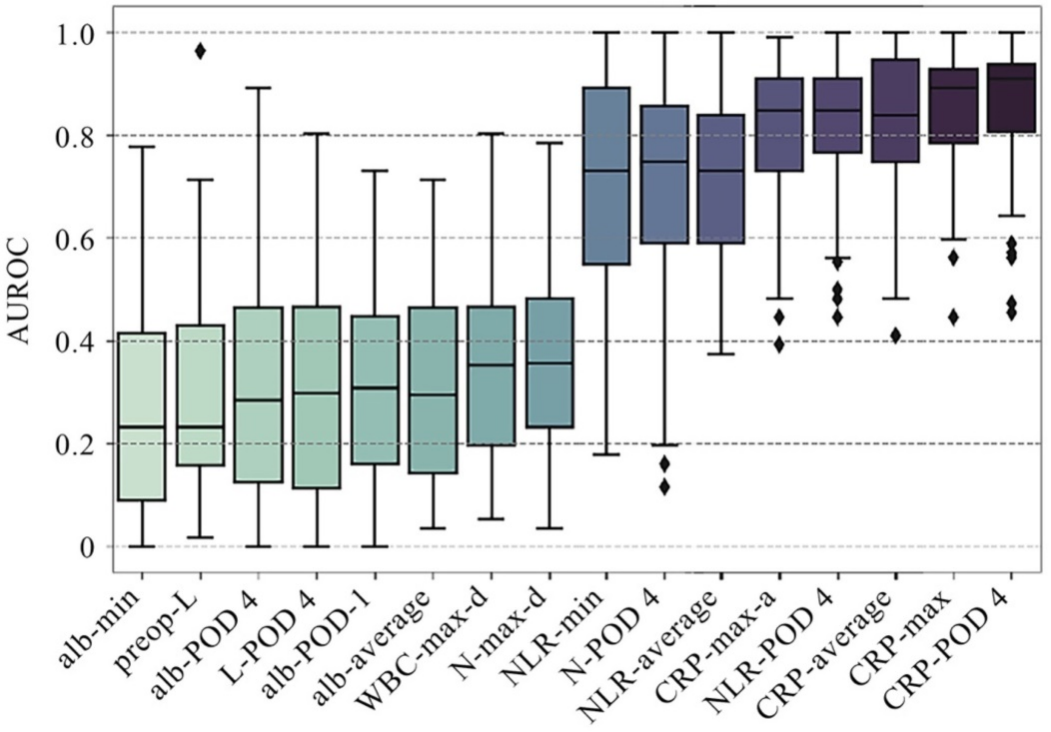
** Comparison of performance of univariate analysis in AUROC.** A total of 16 single factors with top classification ability were presented in the plot, AUROCs of which were presented in median ± standard deviation. AUROCs less than 0.5 correspond to negative correlation with the occurrence of postoperative AL. Average: the average value of the attached clinical variables, including pre-operative and postoperative measurements. Min/max: the minimum/maximum value of the attached clinical variables, including pre-operative and postoperative measurements. Max-a/d: the maximum ascending/descending value of the attached clinical variables between two adjacent measurements. WBC: white blood cell; CRP: C-reactive protein; NLR: neutrophil-to-lymphocyte ratio; PNI: prognostic nutritional index; SII: systemic immune-inflammatory index.

As shown in Table 2a, the median pre-operative CRP, NLR and SII of patients with postoperative AL were significantly higher than those without the occurrence of postoperative AL (P<0.05). While, the median pre-operative lymphocyte count was significantly lower than those without postoperative AL (P<0.05). Among these four preoperative clinical variables that presented statistical difference, lymphocyte count had the best AUC of 0.712 for postoperative AL, which, however, was not significantly higher than that of other preoperative variables. As for postoperative variables, CRP and albumin level on POD 1, as well as all variables on POD 4 showed significant difference between patients with and without AL. Among these variables, CRP on POD 4 achieves optimal AUROC of 0.857 with a cutoff value of 159.5 (Figure 2). However, comparing to preoperative lymphocyte, CRP on POD 4 showed no significant improvement in AUROC (P=0.11).

### Overview of the machine learning classifiers

In order to further determine the predictive value of ML models and immunonutritional index on postoperative AL, we selected 5 machine learning algorithms [*k*-nearest neighbors, logistic regression (LR), support vector machine (SVM), random forest (RF), and gradient tree boosting (GB)] and 6 feature sets, namely nutritional, inflammatory, immunonutritional, all data, all pre-operative data and feature sets with selected variables. The specific variables included in these feature sets respectively were listed in Table 5. Given the fact that many clinical variables in this study were longitudinal (CRP, albumin, etc.), we extracted several features from these variables, including mean value, maximum and minimum account during hospitalization, maximum increase and decrease between adjacent examination, and put these variational indexes into relevant feature sets. The performance of each feature set was listed in Table 4. It is rather clear from the table that LR, SVM and RF models had the first-tier performance, while GB and *k*-NN models had suboptimal performance. Among those three models with first-tier performance, RF model showed the highest AUROCs in most of feature sets, while SVM showed optimal performance in feature set with selected variables, which was also the optimal performance in the entire study (AUROC=0.89±0.09).

### SVM model on selected immunonutritional index had best performance

**Figure 3:**
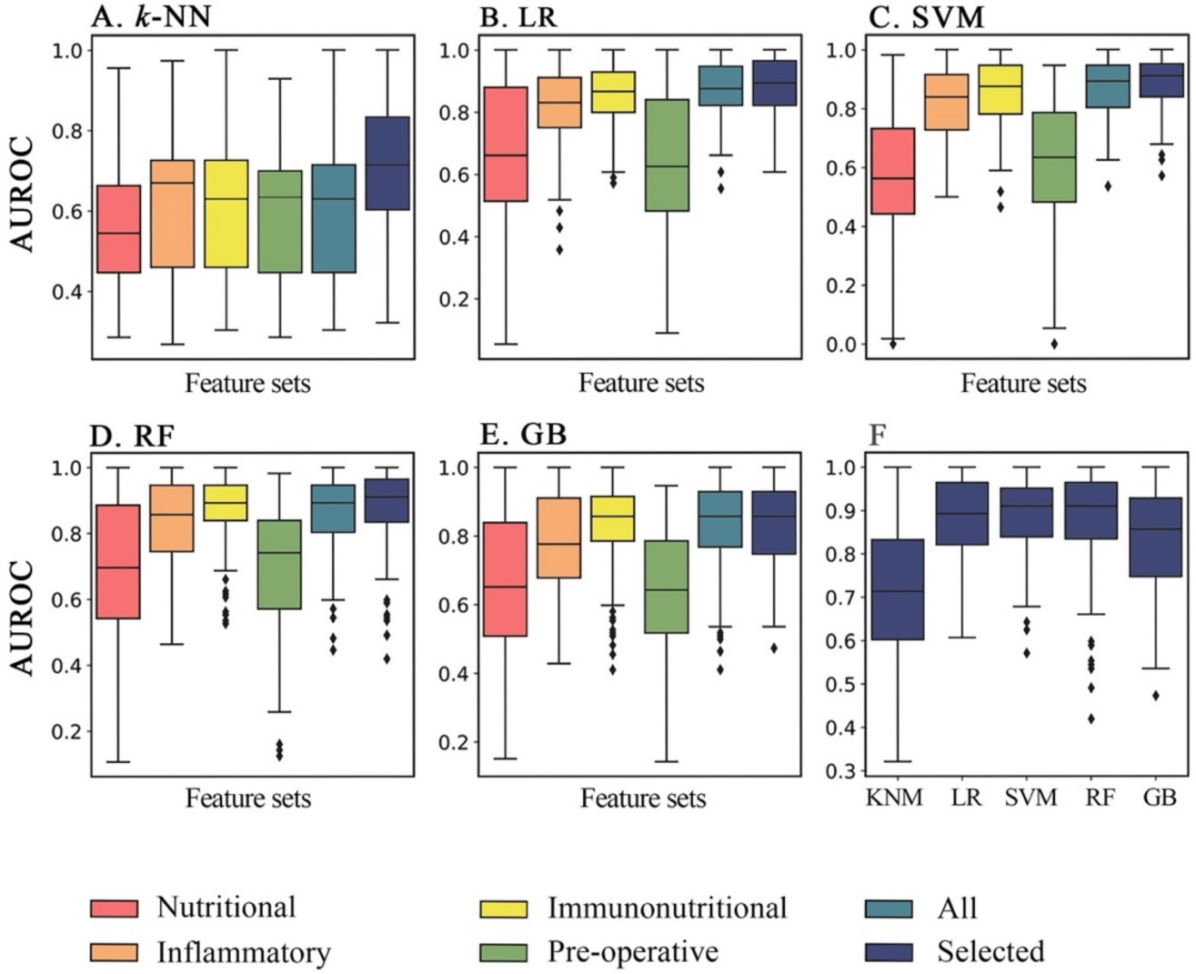
** Comparison of performance of each machine learning methods on 6 feature sets in AUROC.** Overall performance of AUROC (area under the receiver operating characteristic curve) of five machine learning methods for six feature sets respectively in 100 iterations. **A through C,** in *k*-NN, LR and SVM models, the feature set with selected data achieved significant higher classification ability than other 5 remaining feature sets (P<0.05). **D,** as for RF model, the classification ability of selected feature set was optimal, and was significantly higher than that of other feature sets (P<0.05), except for immunonutritional feature set (P=0.078). **E,** in GB model, the selected, immunonutritional and all feature sets showed no significant differences in model performance from one another. Meanwhile, the classification ability of the forementioned 3 feature sets were significantly higher than the remaining 3 feature sets (P<0.05). *k*-NN: *k*-nearest neighbors; LR: logistic regression; SVM: support vector machine; RF: random forest; GB: gradient tree boosting; AUROC: area under the receiver operating characteristic curve

Furthermore, we evaluated the performance of different ML classification models on different feature sets for predicting postoperative AL. According to the analysis, we found that inflammatory or nutritional index alone could achieve a rather promising predictive effect, and that inflammatory index had better overall predictive performance comparing to nutritional index (P<0.01 in all 5 methods). While, combining both forementioned feature sets together with other systemic immunonutritional index like SII and PNI synergistically could further improved the classification performance of the models. As shown in the Figure 3, AUROC of immunonutritional index was significantly higher than that of inflammatory or nutritional feature sets individually in LR, SVM, RF and GB models (P=0.01 for RF, and P<0.01 for other 3 models). Then, we found that *k*-NN, LR and SVM model significantly outperformed themselves in feature set with selected immunonutritional index than other 5 feature sets respectively (P<0.01).

Next, in order to clarify whether or not ML models could improve classification ability on predicting postoperative AL, we picked out and then compared the optimal performance with and without ML models (i.e., SVM model on feature set with selected immunonutritional index and CRP on POD 4, respectively). The result showed that classification ability with ML model was significantly higher than that without ML model (AUROC=0.892 versus 0.857, P=0.02), while the stability of performance with ML model was also significantly higher than that without ML model (P=0.038, Levene test).

**Figure 4:**
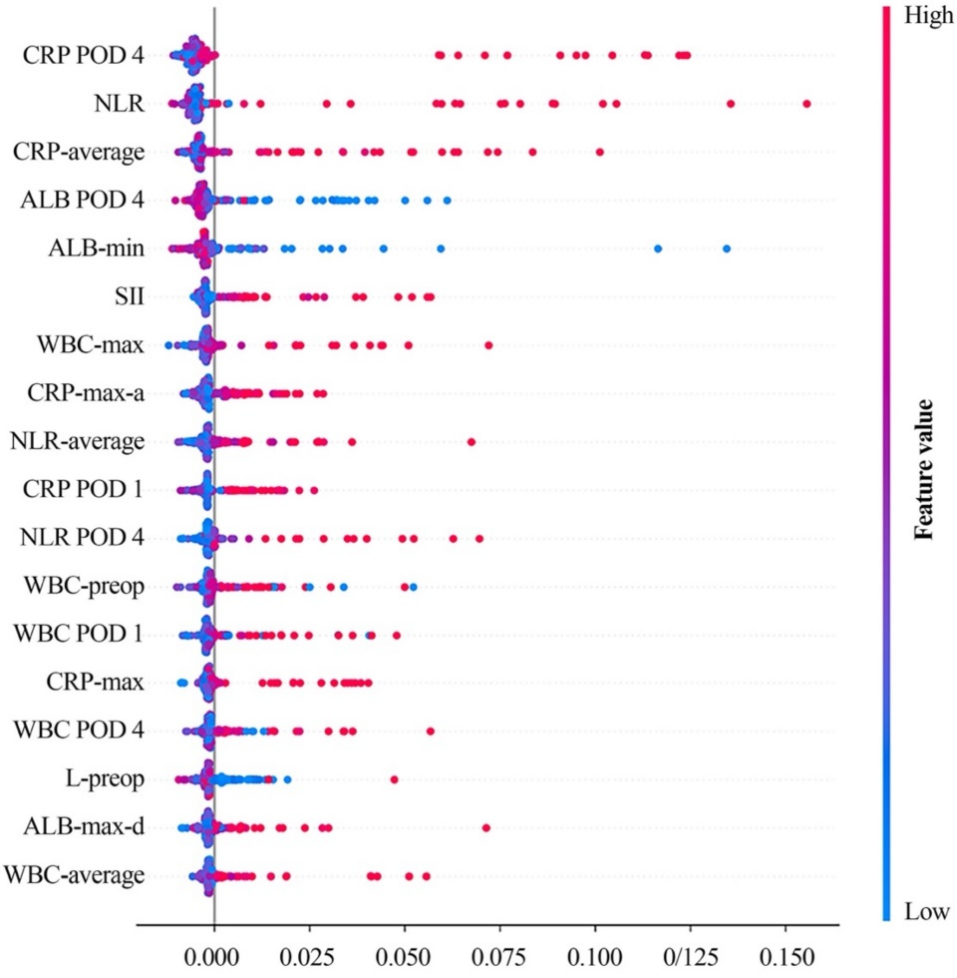
** SHAP value of SVM on selected feature set.** Color gradient indicates that the risk for postoperative AL increases (red) or decreases (blue) as the value of the variables increase. Average: the average value of the attached clinical variables, including pre-operative and postoperative measurements. Min/max: the minimum/maximum value of the attached clinical variables, including pre-operative and postoperative measurements. Max-a/d: the maximum ascending/descending value of the attached clinical variables between two adjacent measurements. SHAP: Shapley additive explanation; SVM: support vector machine; CRP: C-reactive protein; POD 1: postoperative day 1; POD 4: postoperative day 4; NLR: neutrophil-to-lymphocyte ratio; ALB: albumin; SII: systemic immune-inflammatory index; WBC: white blood cell; L: lymphocyte.

After that, we further examined the clinical relevance of different variables in SVM model on feature set with selected variables. As a result, we found that CRP on POD 4 contributed the most to model performance, followed by preoperative NLR, albumin level on POD 4, preoperative SII value etc. (Figure 4).

## Discussion

In this retrospective study of 297 patients from Department of general surgery in Xinhua Hospital, we found that postoperative anastomotic leakage occurred in 6.1% of patients enrolled in this study, which was consistent with previous studies 11, 22. Although, this incidence rate may be slightly higher than the actual situation, since patients with excellent postoperative recovery were more likely to be discharged from hospital early and, therefore, get excluded from the study because of incomplete postoperative variables. From this study, the median postoperative time until diagnosis of AL was 7 days (range 3-30 days), which was also in accordance with present study 11. Thus, with the widespread consensus of ERAS protocol, patients may develop symptoms of postoperative AL after discharge from hospital, arising great clinically concerning risk for prognosis and curative efficacy. Also in this study, patients with postoperative AL were more likely to have prolonged hospital stay and greater amount of intra-operative bleeding, suggesting a worse overall prognosis. So, apparently, it is important to find early and reliable markers for predicting postoperative AL, so that sequelae of severe consequences could be minimized to the most.

Over the years, there were constant discussions on theories for factors of postoperative AL. Some of the factors were accepted by most researchers, including malnutrition, hyper-tensility on the anastomotic stoma, lack of blood supply or local inflammation around anastomotic regions 8, 11, 23. Other possible factors include advanced ages, high BMI, medical history of neoadjuvant chemotherapy, low hemoglobin 7, 22. Apart from the factor of tensility on anastomotic stoma, other involving factors all could be partly summarized into poor immunonutritional condition. Therefore, indexes for describing systemic immunonutritional condition gradually arouse attention of researchers worldwide. Besides inflammatory and nutritional variables, comprehensive immunonutritional index like NLR, SII and PNI also gathered accumulating interest 24-27, receiving promising results for predicting postoperative complication including AL.

From the univariant analysis in this study, we found that CRP on POD 4 achieved optimal AUROC of 0.857 with a cutoff value of 159.5 (Figure 2). This result echoed afar with numerous researches recently 27-31, suggesting that CRP was one of the most commonly used and widely verified clinical predictors for postoperative AL and other postoperative infections. Other variables presenting great potential include NLR on POD 4 (AUROC=0.802, cutoff value=10.55), minimum serum albumin during hospitalization (AUROC=0.7475, cutoff value=32.25) (Figure 2), which also indicated that poor overall immunonutritional internal environment may increase risk of postoperative AL. However, univariant analysis seemed inadequate for analyzing data with complex correlations or processing nonlinear datasets, since both inflammatory and nutrition index needed to be included into analysis.

Therefore, we conducted further research with different machine learning models and feature sets. By comparing the results, we found that both inflammatory variables and nutrition variables could get decent results of predictive ability. While, feature set with inflammatory variables alone could achieve better model performance, with 3 models achieving AUROC > 0.8 and 1 model achieving AUROC > 0.75 (Figure 3). Furthermore, after combining immunonutritional variables, 4 machine learning models showed significant improvement in model performance. (Table 4, P=0.01 for RF, and P<0.01 for other 3 models), which reflected the potential predictive value of immunonutritional indexes. To push even further, we selected a specific set of variables from immunonutritional feature set based on their clinical implications and univariant analysis outcomes. In this way, we set up a more model-specific selection of clinical data to reduce the risk of overfitting. As a result, significant improvement arose in 3 models (Table 4, P<0.01 for *k*-NN, SVM and LR model), and SVM model achieved optimal AUROC of 0.89.

**Figure 5:**
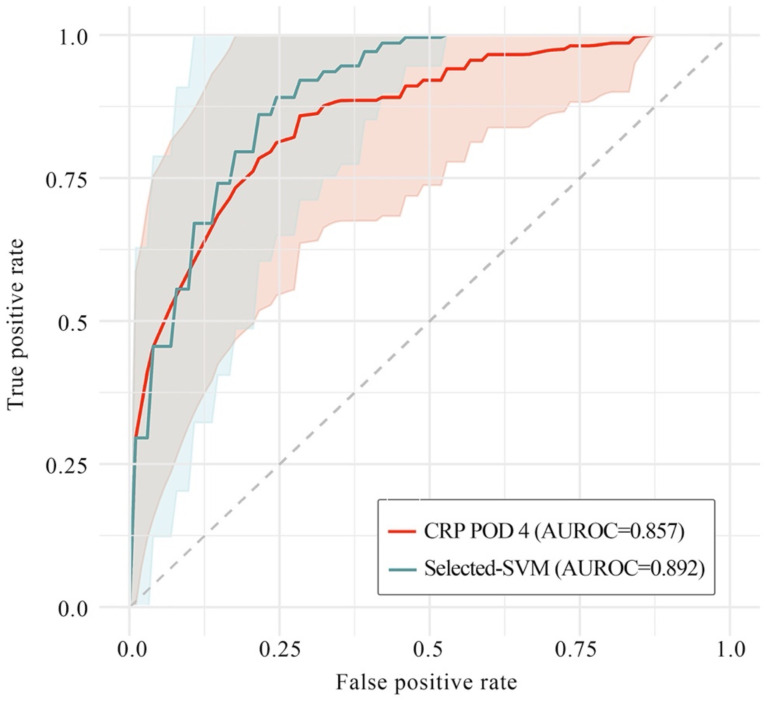
** Performance of SVM model and CRP on postoperative day 4 in AUROC.** In this plot, the saturated-colored lines are the average ROCs of 100 iteration respectively, while the light-colored area correspond to mean ± standard deviation. The grey dotted line is the baseline of a random classifier. The average AUROCs were presented respectively in this plot. SVM model on selected feature set outperformed CRP on POD 4 (P=0.02), while the stability of SVM was also significantly higher than CRP on POD 4 (P=0.038, Levene test). SVM: support vector machine; CRP: C-reactive protein; POD 4: postoperative day 4; AUROC: area under the receiver operating characteristic curve.

Then, naturally, we needed to find out if ML models could significantly improve the classification ability on predicting postoperative AL. In order to figure out that, we picked out the optimal performance with ML model (i.e., SVM model on feature sets with selected immunonutritional index) and the optimal performance without ML (i.e., CRP on POD 4). Then, through comparison between these two performances, we found out that classification ability with. ML was significantly higher than that without ML (Figure 5, P=0.02). Moreover, the stability of the performance with ML was also significantly higher (P=0.038). The result showed that ML models presented valid advantages over common univariant analysis, which also indicated that the occurrence of postoperative AL involves multiple factors contributing collectively. To understand which variables contributed to the model performance, we examined the learning weight of the feature sets (Figure 4). According to the plot of SHAP value, we found that increased serum CRP level and decreased albumin level on POD 4 were associate with high risk of postoperative AL. Other variables with clinically relevance included NLR, white blood cell counts and SII (Figure 4).

Furthermore, the study results also suggested that pre-operative variables alone were inadequate for accurate prediction (Table 4, Figure 3). As for pre-operative risk factors, some researchers found that pre-operative radiotherapy [OR = 1.65 (95% CI: 1.06-2.56)] and gender male [OR = 1.48 (95% CI: 1.37-1.60)] could be regarded as risk factors for postoperative AL, but the quality of evidence was moderate to low according to the GRADE approach 32, which, in some way, was in accordance with the outcome in this study.

Additionally, we found that variational index also contributed greatly in the model (Figure 4), like minimum albumin level, maximum count of white blood cell and maximum ascending amount of CRP etc. Several studies have learnt the importance of trajectory of clinical variables in prognostic research 33, 34, although some of the research also suggested that variational index alone lacked predictive value to rule out postoperative AL 35. In this study, we combined variational index with other perioperative variables and examined by machine learning methods, and eventually found the clinical contribution and importance of the changes in these variables.

In this study, we found that among 5 machine learning methods mentioned above, RF achieved the best performance in most feature sets, while LR, SVM and RF all reached first tier performance on feature set of selected variables, showing no significant difference from one another. This result suggested that machine learning models could extract useful information more effectively from feature sets with selected data, due to the removal of redundant data in the feature set.

Another thing to be noticed, we took records of clinical variables on POD 7 as mentioned above. As a result, CRP on POD 7 stood out with AUROC of 0.930, which had the optimal classification ability comparing with any other variables or feature sets on machine learning models. Consequently, it seemed that whether or not applying machine learning models made no difference to final performances. Should it be solid, CRP on POD 7 would be the solely crucial factor for AL, which seemed clinically unlikely to be true. To further identify the possible underlying reasons behind this outcome, we re-evaluated the clinical details of all the 18 patients with postoperative AL, finding that nearly half of these patients (n=8) presented symptoms of AL within POD 7 (Table 1). This could indicate that, for these patients, CRP on POD 7 should be considered as the outcome of postoperative AL instead of risk factor. Since patients with postoperative AL were already in a small amount, we decided that CRP on POD 7 should not be applied in the study. Recent studies also showed that researchers tend to conduct blood tests on postoperative day 1, 3, 4 or 5 10, 20, 29, 36. Besides that, another limitation of this study lied in the lack of cases in the study, especially cases of patients with postoperative AL, causing unstable performance of the models. Also, the negative predictive values of the models were generally above 0.90, which could be a result of a relatively low incidence of postoperative AL. This is another reason for the lack of cases of AL.

Thus, our future research will focus on further expanding the dataset, especially the collection of patients with postoperative AL, and standardizing the collection time points of laboratory examination. Apart from that, we will also integrate this machine learning-based risk assessment with online tools for clinical practice. For example, a combination of dataset and online risk calculator could enable real-time risk assessment while clinical data being recorded.

## Conclusion

In the study, machine learning models were built for risk assessment of postoperative anastomotic leakage with 6 feature sets and 5 classification methods. We found that immunonutritional index have moderate to high performances, and selected index could further improve the classification ability of the model. We found that ML models could significantly improve classification ability than common univariant analysis. We also identified several variables with clinical relevance to postoperative AL, providing potential biomarkers for postoperative healthcare. This study indicated that machine learning-based risk assessment with the help of immunonutritional index could be a useful tool for early detection and decision-making for clinical practice in gastric cancer treatment.

## Publication ethnics

This study was conducted with informed consent of all participants. All patient data were de-identified.

## Figures and Tables

**Table 2a:** Pre-operative and overall clinicopathological characteristics of the study stratified with and without post-operative anastomotic leakage (n=297)

Variables	All patients (n=297)	Post-operative anastomotic leakage		P-value
		No (n=279)	Yes (n=18)	
Sex				0.583
Female	98 (33%) ^*^	91 (32.6%)	7 (38.9%)	
Male	199 (67%)	188 (67.4%)	11 (61.1%)	
Age (years)	65 [57-70] ^†^	65 [57-70]	61 [52.8-69]	0.277
BMI (kg/m^2^)	22.4 ± 3.2	22.5 ± 3.2	21.6 ± 3.7	0.277
Neoadjuvant chemotherapy				0.357
No	290 (97.6%)	273 (97.8%)	17 (94.4%)	
Yes	7 (2.4%)	6 (2.2%)	1 (5.6%)	
Preoperative WBC count ( × 10^9^/L)	5.6 [4.6-6.8]	5.6 [4.7-6.8]	5.6 [4.2-8]	0.814
Preoperative CRP	2 [1-3]	2 [1-3]	3 [2-5.5]	0.009
Preoperative neutrophil ( × 10^9^/L)	3.4 [2.7-4.3]	3.3 [2.7-4.2]	4.3 [2.9-5.4]	0.065
Preoperative lymphocyte ( × 10^9^/L)	1.6 [1.2-2]	1.6 [1.3-2]	1.2 [1-1.4]	0.003
Preoperative hemoglobin (g/L)	129 [111-140]	129 [111-140]	123 [113-146.8]	0.976
Preoperative albumin (g/L)	39.4 ± 4.6 ^#^	39.5 ± 4.5	38.1 ± 5.3	0.228
Preoperative NLR	2.2 [1.6-2.9]	2.1 [1.5-2.9]	3.1 [2.2-4.8]	0.003
Preoperative PNI	47.7 ± 5.9	47.8 ± 5.8	45.1 ± 6.5	0.056
Preoperative SII	436.8 [295.6-706.1]	435 [295.4-691.8]	828.4 [402.9-1077.6]	0.029
Post-operative hospital stays (days)	11 [9-13]	11 [9-13]	27.5 [21.2-33.5]	< 0.001
Clavien-Dindo classification				< 0.001
I	245 (82.5)	245 (87.8)	0 (0)	
II	37 (12.5)	33 (11.8)	4 (22.2)	
III	12 (4)	1 (0.4)	11 (61.1)	
IV	2 (0.7)	0 (0)	2 (11.1)	
V	1 (0.3)	0 (0)	1 (5.6)	

^*^ Categorical variables are presented as number (percentage). ^#^ Continuous variables are presented as mean ± standard deviation for normally distributed data. ^†^ Other continuous are presented as medians and [interquartile ranges]. BMI: Body Mass Index; WBC: white blood cell; CRP: C-reactive protein; NLR: neutrophil-to-lymphocyte ratio; PNI: prognostic nutritional index; SII: systemic immune-inflammatory index.

**Table 2b:** Operative and tumor-related clinicopathological characteristics of the study stratified with and without post-operative anastomotic leakage (n=297)

Variables	All patients (n=297)	Post-operative anastomotic leakage		P-value
		No (n=279)	Yes (n=18)	
Intraoperative blood loss (mL)	150 [100-200]	150 [100-200]	200 [112.5-325]	0.042
Intra-operative blood transfusion:				0.478
No	260 (87.5%)	245 (87.8%)	15 (83.3%)	
Yes	37 (12.5%)	34 (12.2%)	3 (16.7%)	
Surgical approach:				0.565
Laparotomy	196 (66%)	183 (65.6%)	13 (72.2%)	
Laparoscopic or laparoscopic assisted	101 (34%)	96 (34.4%)	5 (27.8%)	
TNM stage				0.572
I	102 (34.3%)	98 (35.1%)	4 (22.2%)	
II	67 (22.6%)	63 (22.6%)	4 (22.2%)	
III	124 (41.8%)	114 (40.9%)	10 (55.6%)	
IV	4 (1.3%)	4 (1.4%)	0 (0)	

The tumor staging was cataloged based on the American Joint Committee on Cancer of Gastric Cancer TNM staging system (eighth edition).

**Table 2c:** Postoperative clinicopathological characteristics of the study stratified with and without post-operative anastomotic leakage (n=297)

Variables	All patients (n=297)	Post-operative anastomotic leakage		P-value
		No (n=279)	Yes (n=18)	
WBC-POD 1	14.2 [11.3, 17.5]	14.1 [11.3, 17.2]	16.3 [10.3, 20.9]	0.366
CRP-POD 1	72 [47, 99]	69 [47, 98]	125.5 [58.2, 149.2]	0.02
N-POD 1	12.5 [9.9, 15.8]	12.4 [9.9, 15.7]	14.9 [9.2, 19.4]	0.359
L-POD 1	0.7 [0.6, 0.9]	0.7 [0.6, 1]	0.7 [0.4, 0.8]	0.279
ALB-POD 1	35.6 ± 4.4	35.9 ± 4.2	31.6 ± 5.8	< 0.001
NLR-POD 1	17.5 [11.6, 24.7]	17.4 [11.6, 24.6]	20.1 [13.6, 26.6]	0.277
WBC-POD 4	10.1 [7.6, 12.3]	10 [7.5, 12.1]	11.8 [10.2, 16]	0.02
CRP-POD 4	89 [54, 157]	85 [52, 145.5]	180 [160, 200]	< 0.001
N-POD 4	8.3 [6, 10.4]	8.2 [5.9, 10]	10.3 [8.8, 13.4]	0.005
L-POD 4	0.9 [0.7, 1.3]	0.9 [0.7, 1.3]	0.8 [0.5, 1]	0.015
ALB-POD 4	36.9 ± 4	37.1 ± 3.9	34 ± 4.6	0.001
NLR-POD 4	8.4 [5.9, 12.4]	8.2 [5.7, 12]	14.2 [12.1, 23.4]	< 0.001

WBC: white blood cell; CRP: C-reactive protein; NLR: neutrophil-to-lymphocyte ratio; N: neutrophil; L: lymphocyte; alb: albumin; POD: postoperative day.

**Table 3:** Value of hyperparameters of classification methods

Classifier	Hyperparameter	Value
k-nearest neighbor	n-neighbors	3, 5, 7
	metrics	Euclidean, correlation
Logistic regression	C	0.01, 0.1, 1, 10,100, 1000
Support vector machine	C	0.1, 1, 10, 100
	gamma	1e-3, 1e-2, 1e-1
Random forest	Max features	0.1, 0.2, 0.4, 0.8
	Max depth	4, 8, 12
Gradient tree boosting	N estimators	100, 500
	Max depth	2, 3, 4
	Learning rate	0.01, 0.05, 0.1
	Subsample	0.33, 0.66, 1

**Table 4:** AUROC for each machine learning model on 6 feature sets

Feature sets	*k*-NN	LR	SVM	RF	GB
Pre-operative	0.57±0.15	0.63±0.24	0.61±0.21	0.69±0.2	0.64±0.19
Inflammatory	0.63±0.17	0.81±0.13	0.81±0.13	0.83±0.14	0.78±0.15
Nutritional	0.55±0.14	0.67±0.23	0.56±0.23	0.68±0.22	0.65±0.22
Immunonutritional	0.62±0.17	0.85±0.1	0.85±0.12	0.87±0.12	0.82±0.14
All	0.61±0.18	0.87±0.09	0.87±0.1	0.86±0.13	0.82±0.14
Selected	0.71±0.17	0.88±0.09	0.89±0.09	0.87±0.13	0.83±0.13

AUROC (area under the receiver operating characteristic curve) was presented in mean ± standard deviation for each machine learning model on 6 feature sets in this study. *k*-NN: *k*-nearest neighbors; LR: logistic regression; SVM: support vector machine; RF: random forest; GB: gradient tree boosting

**Table 5:** List of clinical variables included in each feature set

	Nutri- ional	Inflamma-tory	Immuno- nutritional	All	Pre- operative	Selected	
Sex	0	0	0	1	1	0	
Age	0	0	0	1	1	1	
Height	0	0	0	1	1	0	
Weight	0	0	0	1	1	0	
BMI	1	0	1	1	1	1	
Neoadjuvant chemotherapy	0	0	0	1	1	0	
Diabetes mellitus	0	0	0	1	1	1	
Hypertension	0	0	0	1	1	0	
preop-WBC	0	1	1	1	1	1	
preop-CRP	0	1	1	1	1	1	
preop-N	0	1	1	1	1	0	
preop-L	0	1	1	1	1	1	
preop-blood platelet	1	0	1	1	1	0	
preop-hemoglobin	1	0	1	1	1	1	
preop-alb	1	0	1	1	1	1	
NLR	0	1	1	1	1	1	
PNI	1	0	1	1	1	1	
SII	0	1	1	1	1	1	
Preoperative fibrinogen	1	0	1	1	1	0	
op-bleed	0	0	0	1	0	1	
WBC-POD 1/4	0	1	1	1	0	1	
CRP-POD 1/4	0	1	1	1	0	1	
N-POD 1/4	0	1	1	1	0	0	
L-POD 1/4	0	1	1	1	0	0	
ALB-POD 1/4	1	0	1	1	0	1	
NLR-POD 1/4	0	1	1	1	0	1	
N-max	0	1	1	1	0	0	
N-min	0	1	1	1	0	0	
N-max-a	0	1	1	1	0	0	
N-max-d	0	1	1	1	0	0	
N-average	0	1	1	1	0	0	
WBC-max	0	1	1	1	0	1	
WBC-min	0	1	1	1	0	0	
WBC-max-a	0	1	1	1	0	1	
WBC-max-d	0	1	1	1	0	0	
WBC-average	0	1	1	1	0	1	
NLR-max	0	1	1	1	0	1	
NLR-min	0	1	1	1	0	0	
NLR-max-a	0	1	1	1	0	1	
NLR-max-d	0	1	1	1	0	0	
NLR-average	0	1	1	1	0	1	
CRP-max	0	1	1	1	0	1	
CRP-min	0	1	1	1	0	0	
CRP-average	0	1	1	1	0	1	
CRP-max-a	0	1	1	1	0	1	
CRP-max-d	0	1	1	1	0	0	
alb-max	1	0	1	1	0	0	
alb-min	1	0	1	1	0	1	
alb-average	1	0	1	1	0	1	
alb-max-a	1	0	1	1	0	0	
alb-max-d	1	0	1	1	0	1	

WBC: white blood cell; CRP: C-reactive protein; NLR: neutrophil-to-lymphocyte ratio; PNI: prognostic nutritional index; SII: systemic immune-inflammatory index, alb: albumin; POD: postoperative day; preop: preoperative; N: neutrophil; L: lymphocyte. Average: the average value of the attached clinical variables, including pre-operative and postoperative measurements. Min/max: the minimum/maximum value of the attached clinical variables, including pre-operative and postoperative measurements. Max-a/d: the maximum ascending/descending value of the attached clinical variables between two adjacent measurements.

**Table 1:** Details of patients with postoperative anastomotic leakage

	Sex	Age	BMI	Antecedents	Diagnostic delay	Clinical signs	Clavien-Dindo classification	NLR (Preop-POD1-4-7)	CRP (Preop-POD1-4-7)	Clinical outcome
1	F	74	16.22	DM	D6	Fever	IIIb	3.60 -35.53 -49.22 -23.18	24-143-160-132	Recovered on POD 36
2	M	50	21.63	Neoadjuvant chemotherapy (FLOT)	D16	Epigastric pain; unconsciousness	IVa	6.40 -36.34 -5.36 -8.32	2-160-50-48	Recovered on POD 45
3	M	59	20.42	Hypertension	D8	Fever	IIIa	2.17 -25.23 -24.98 -21.08	4-160-160-160	Recovered on POD 25
4	F	61	17.42	Hypertension	D30	Epigastric pain	IIIa	4.21 -14.13 -10.61 -3.23	3-124-129-99	Recovered on POD 51
5	F	41	19.40	/	D7	Fever	II	3.83 -19.57 -12.57 -3.80	1-33-85-99	Recovered on POD 28
6	M	72	17.36	Hypertension	D8	Ventosity; abdominal pain	V	5.38 -37.00 -9.33- 18.80	4-68-114-160	Death on POD11
7	M	69	25.71	Sequelae of previous cerebral infarction	D7	Abdominal pain	III	5.50 -50.25 -39.17 -42.63	3-160-200-200	Recovered on POD 31
8	M	71	22.77	Tobacco; COPD	D3	Tachycardia	IV	1.82 -13.88 -30.03 -65.00	2-78-200-200	Recovered on POD 32
9	M	51	26.92	Hypertension; DM	D5	Jaundice; abdominal pain	III	4.77 -13.40 -16.35 -8.06	3-32-200-200	Recovered on POD 29
10	M	55	22.84	/	D16	Fever	III	1.10 -11.15 -8.17 -4.04	3-160-200-160	Recovered on POD 40
11	F	76	17.22	Osteoporosis; hypertension; DM	D12	Increased drainage	II	4.82 -23.79 -12.57 -15.33	6-150-200-200	Recovered on POD 31
12	M	46	24.98	Tobacco	D9	Epigastric pain	II	2.65 -16.67 -14.42 -20.62	1-48-200-200	Recovered on POD 27
13	M	52	24.38	Tobacco	D3	Fever	IIIa	2.43 -20.74 -14.38 -6.20	2-55-160-160	Recovered on POD 20
14	F	55	25.78	/	D5	Hemorrhagic drainage	IIIb	2.48 -27.09 -27.93 -8.26	1-91-200-200	Recovered on POD 34
15	M	61	23.70	Hypertension; asthma	D8	Fever	IIIa	2.21 -11.63 -13.59 -11.35	6-133-200-160	Recovered on POD 28
16	M	69	18.38	Hypertension; DM	D8	Purulency drainage	IIIa	4.91 -13.50 -18.60 -12.84	8-147-200-200	Recovered on POD 31
17	F	66	17.57	/	D5	Epigastric pain	II	1.94 -5.91 -11.96 -11.92	30-127-160-160	Recovered on POD 18
18	F	68	26.35	Hypertension	D8	Epigastric pain; fever	III	5.61 -20.67 -14.00 -10.70	1-33-160-96	Recovered on POD 37

FLOT: Fluorouracil, Leucovorin, Oxaliplatin and Docetaxel; DM: Diabetes mellitus; COPD: chronic obstructive pulmonary disease; BMI: Body Mass Index; NLR: neutrophil-to-lymphocyte ratio; POD postoperative day.

## Abbreviations

*k*-NN: *k*-nearest neighbors
LR: logistic regression
SVM: support vector machine
RF: random forest
GB: gradient tree boosting
AUROC: area under the receiver operating characteristic curve
SHAP: Shapley additive explanation
CRP: C-reactive protein
POD: postoperative day
NLR: neutrophil-to-lymphocyte ratio
PNI: prognostic nutritional index
ALB: albumin
SII: systemic immune-inflammatory index
WBC: white blood cell
L: lymphocyte
N: neutrophil
FLOT: Fluorouracil, Leucovorin, Oxaliplatin and Docetaxel
DM: Diabetes mellitus
COPD: chronic obstructive pulmonary disease
BMI: Body Mass Index
